# Altered Development of the Hurst Exponent in the Medial Prefrontal Cortex in Preschoolers With Autism

**DOI:** 10.1016/j.bpsc.2024.09.003

**Published:** 2024-09-16

**Authors:** Annika C. Linke, Bosi Chen, Lindsay Olson, Michaela Cordova, Molly Wilkinson, Tiffany Wang, Meagan Herrera, Madison Salmina, Adriana Rios, Judy Mahmalji, Tess Do, Jessica Vu, Michelle Budman, Alexis Walker, Inna Fishman

**Affiliations:** Department of Psychology, San Diego State University, San Diego, California (ACL, MH, MS, AR, JM, TD, JV, MB, AW, IF); San Diego State University/University of California San Diego Joint Doctoral Program in Clinical Psychology, San Diego, California (ACL, BC, LO, MC, MW, IF); Center for Autism and Developmental Disorders, San Diego State University, San Diego, California (ACL, IF); Department of Child and Adolescent Psychiatry, New York University School of Medicine, New York, New York (BC); Department of Psychiatry and Behavioral Sciences, University of California San Francisco, San Francisco, California (LO); and Department of Psychology, University of California San Diego, La Jolla, California (TW).

## Abstract

**BACKGROUND:**

Atypical balance of excitation (E) and inhibition (I) in the brain is thought to contribute to the emergence and symptomatology of autism spectrum disorder (ASD). E/I ratio can be estimated from resting-state functional magnetic resonance imaging (fMRI) using the Hurst exponent, *H*. A recent study reported decreased ventromedial prefrontal cortex (vmPFC) *H* in male adults with ASD. Part of the default mode network (DMN), the vmPFC plays an important role in emotion regulation, decision making, and social cognition. It frequently shows altered function and connectivity in individuals with autism.

**METHODS:**

The current study presents the first fMRI evidence of altered early development of vmPFC *H* and its link to DMN functional connectivity and emotional control in toddlers and preschoolers with ASD. A total of 83 children (45 with ASD), ages 1.5–5 years, underwent natural sleep fMRI as part of a longitudinal study.

**RESULTS:**

In a cross-sectional analysis, vmPFC *H* decreased with age in children with ASD, reflecting increasing E/I ratio, but not in typically developing children. This effect remained significant when controlling for gestational age at birth, socioeconomic status, or ethnicity. The same pattern was also observed in a subset of children with longitudinal fMRI data acquired 2 years apart on average. Lower vmPFC *H* was also associated with reduced functional connectivity within the DMN as well as with higher emotional control deficits (although only significant transdiagnostically).

**CONCLUSIONS:**

These results suggest an early onset of E/I imbalances in the vmPFC in ASD, with likely consequences for the maturation of the DMN.

Neuroimaging studies of autism spectrum disorder (ASD) over the past decades have revealed that ASD is characterized by distributed functional brain network abnormalities that have been documented across the entire life span (1–4). However, the neuronal mechanisms that underlie atypical brain function and its development in ASD remain unknown. Atypical balance of excitation (E) and inhibition (I) in the brain (through altered glutamatergic and GABAergic [gamma-aminobutyric acidergic] neurotransmission, respectively) is thought to contribute to the emergence and symptomatology of neurodevelopmental disorders [e.g., (5,6)]. In ASD, evidence of E/I imbalance comes from postmortem and positron emission tomography studies showing reduced numbers of GABA receptors and prefrontal GABAergic parvalbumin interneurons (7,8); from magnetic resonance spectroscopy showing reduced GABA levels in frontal and temporal lobes, sensorimotor cortex and the cerebellum (9,10); from human brain organoid and animal models of autism [for reviews, see (11,12)]; and more indirectly from the increased prevalence of epilepsy and epileptiform electroencephalography (EEG) in ASD [e.g., (13,14)].

Rubenstein and Merzenich (6) suggested that hyperexcit-ability may result in increased aperiodic neural activity (“neural noise”) in autism. It was later shown by Gao *et al*. (15) using simulations and animal models that a flatter 1/*f* slope (i.e., increased aperiodic activity) in electrophysiological time series data seemed to be associated with increased E/I ratio. However, surprisingly few studies have used EEG or magnetoencephalography to derive the power law exponent (i.e., slope) [as in (15)] or closely related Hurst exponent, *H*, which measures the long-range temporal correlation of a time series [see (16,17) for reviews] as a marker for E/I ratio in human studies of autism. Using magnetoencephalography, Manyukhina *et al*. (18) found globally flatter 1/*f* slopes in school-aged children with ASD that were associated with lower IQ. Increased neural noise derived from EEG has also been observed in attention-deficit/hyperactivity disorder (19–21) and schizophrenia (22–24), but the relationship to E/I ratio in humans remains unclear. Recently, Trakoshis *et al*. (25) confirmed Gao *et al*.’s results using more realistic simulations that were validated by in vivo chemogenetic manipulations and simultaneous functional magnetic resonance imaging (fMRI) in mice to directly test the effect on the 1/*f* slope and *H* when the E/I ratio was manipulated. These experiments demonstrated that neural noise can also be reliably quantified from fMRI data and that lower *H* reflects increased E/I ratio. Importantly, in the same study, it was also reported that *H* as derived from resting-state fMRI was reduced in the ventromedial prefrontal cortex (vmPFC) (area p32) in young male adults with ASD compared with a group of neurotypical control participants. *H* was increased in the same region in female adults with ASD and, in females only, was associated with reduced sociocommunicative symptoms. The authors interpreted these results as pointing to an early developmental origin of E/I imbalances in ASD being linked to androgen-sensitive autism risk genes, consistent with the well-documented sex bias in ASD incidence. The vmPFC is part of the default mode network (DMN) and plays an important role in emotion regulation, decision making, and social cognition, with altered function and connectivity consistently reported in children, adolescents, and adults with ASD [see Padmanabhan *et al*. (26) for a review]. Here, we aimed to test whether reduced *H* in the vmPFC could be detected in a cohort of toddlers and preschoolers with ASD who underwent fMRI during natural sleep, presenting the first fMRI evidence of altered development of vmPFC E/I balance and its links to functional connectivity (FC) in early life in ASD.

## METHODS AND MATERIALS

### Participants

A total of 56 young children with ASD and 38 typically developing (TD) children, ages 1.5 to 5 years (15–65 months), completed a natural sleep MRI session as part of a longitudinal study of brain development. Children with a (suspected) diagnosis of autism were referred from specialty autism clinics, state-funded early education and developmental evaluation programs, local pediatricians, service providers, and community clinics; TD children were recruited from the community. ASD diagnoses were established based on DSM-5 at the SDSU Center for Autism and Developmental Disorders, using the Autism Diagnostic Observation Schedule-Second Edition (27), the Autism Diagnostic Interview-Revised (for children older than 36 months) (28), and expert clinical judgment according to the recommendations by the American Academy of Pediatrics and Society for Developmental and Behavioral Pediatrics (29). Parents also completed the Social Communication Questionnaire (30), a screener for ASD, with no TD participants exceeding the cutoff score of 15 (all TD scores ≤ 10) (see the Supplement for further exclusionary criteria). All participants were safety screened for MRI contraindications (see the Supplement for additional exclusionary criteria). Consent was acquired from caregivers, and families were compensated for their time. The research protocol was approved by the institutional review boards of the University of California San Diego and San Diego State University. MRI data from 45 children with ASD and 38 TD children were included in analysis after MRI data quality assessment (see the Supplement; Table 1).

### MRI Data

Natural sleep MRI data were collected at the University of California San Diego Center for Functional MRI using a GE 3T Discovery MR750 scanner and Nova Medical 32-channel head coil. The MRI protocol included two 6-minute multiband echo-planar imaging acquisitions (repetition time = 800 ms, echo time = 35 ms, flip angle 52°, 72 slices, multiband acceleration factor 8, 2-mm isotropic voxel size, 104 × 104 matrix size, field of view = 20.8 cm, 400 volumes per run) and a fast 3-dimensional spoiled gradient recalled T1-weighted sequence (0.8-mm isotropic voxel size, number of excitations = 1, repetition time = 8.776 ms, echo time = 3.656, inversion time = 1060 ms, flip angle 8°, field of view = 25.6 cm, matrix = 320 × 320, receiver bandwidth 31.25 Hz). Motion during structural acquisitions was corrected in real-time using 3 navigator scans [real-time prospective motion correction (31)], and images were bias corrected using the GE PURE option.

MRI data were preprocessed, denoised, and analyzed in MATLAB (version 2017b; The MathWorks, Inc.) using SPM12 and the CONN toolbox version 17f (32). fMRI data were realigned, normalized to Montreal Neurological Institute, bandpass filtered, and underwent nuisance regression to remove physiological noise and motion confounds [see (33) and the Supplement for detailed information on natural sleep MRI, preprocessing, and denoising procedures]. Ten children (7 with ASD, 3 TD) only had 1 fMRI acquisition available for analyses (8 due to high motion in one of the 2 runs or due to waking up prior to the end of both fMRI scans and 2 due to technical malfunction during data collection). For the main analyses, only the lowest motion fMRI run was analyzed to maximize similarity of data quality for the ASD and TD groups (but see Linear Mixed-Effects Model for an additional analysis including all available data to test the robustness of the findings).

### Hurst Exponent Analyses

Average voxel time series were extracted from the preprocessed and denoised fMRI data for the same p32 parcel of the HCP (Human Connectome Project) Multimodal Parcellation (34) as in Trakoshis *et al*. (25). *H* was estimated for the p32 time series with a discrete wavelet transform and fractionally integrated processes model using maximum likelihood estimation as implemented by the bfn_mfin_ml.m function in the nonfractal MATLAB toolbox (https://github.com/wonsang/nonfractal), using the same parameter settings as used by Trakoshis *et al*. (25) (i.e., the ‘haar’ filter option was chosen with [1.5,10] for the upper and [20.5,0] set for the lower bounds).

An analysis of covariance (ANCOVA) was used to test for main effects of diagnostic group (ASD, TD; referred to as group henceforth), sex, and age, and group × sex and group × age interactions in p32 *H*, with motion during the fMRI scan (root mean-square deviation [RMSD]) added as a covariate of no interest. Given the small number of girls in our cohort, an additional analysis was conducted in a male-only sample (see Table S1 for male-only sample characteristics), with an ANCOVA testing for main effects of group and age and group × age interaction, with RMSD added as a covariate. To address potential confounds due to inclusion of prematurely born children in the ASD group (as prematurity constitutes a known risk factor for ASD), a supplementary analysis was also conducted excluding those born <36 weeks gestational age (see Table S2).

### Longitudinal Analyses

Longitudinal fMRI data were available for 15 TD participants and 10 participants with ASD with an average time of 26.3 months between scans (SD = 11.0) (see Table S3 for longitudinal cohort characteristics). A repeated-measures analysis of variance was used to test for main effects of diagnostic group and time point (TP) and a group × TP interaction on vmPFC *H*.

### Linear Mixed-Effects Model

To assess the robustness of the findings while also including the alternative fMRI acquisition (for children with 2 high-quality fMRI runs), all available data (2 runs and/or TPs) were analyzed using a linear mixed-effects model that tested—like the main analysis described in MRI Data—for main effects of group, sex, age, RMSD, as well as group × age and group × sex interactions.

### *H*-FC Associations

Area p32 is part of the DMN. To test the potential impact of differences in vmPFC *H* on the functional organization of the DMN, average FC between area p32 and the regions of the DMN as defined by the HCP network atlas included with the CONN toolbox was calculated. First, the voxel time series in each DMN network region (posterior cingulate cortex, medial prefrontal cortex, and bilateral lateral parietal cortex) were averaged. FC was then calculated as the Fisher *z*-transformed Pearson correlation between the average voxel time series derived from area p32 and that of each DMN network region. These 4 FC estimates were then further averaged to yield a measure of within-DMN FC (with p32 as the seed region). DMN FC served as the dependent variable in an ANCOVA testing for main effects of group and vmPFC *H* and a group × *H* interaction (with RMSD as a covariate of no interest). An additional ANCOVA tested for effects of age and group × age interactions (controlling for RMSD) on FC within the DMN.

### *H*-Behavior Associations

Partial correlations (controlling for RMSD) were used to test for any associations between vmPFC *H* and ASD symptom severity (Autism Diagnostic Observation Schedule-Second Edition comparison scores) in the ASD group. Because the vmPFC plays an important role in emotional regulation, the association between vmPFC *H* and the emotional control subscale of the Behavior Rating Inventory of Executive Function (BRIEF)-Preschool Version (35) completed by caregivers was also assessed (partial correlation controlling for RMSD). Since the BRIEF-Preschool was only administered to a subset of children (20 with ASD, 14 TD), this correlation was assessed across groups.

## RESULTS

### Different Developmental Trajectory of vmPFC *H* in ASD

Results of the ANCOVA revealed a significant group × age interaction effect on *H*, such that in toddlers and preschoolers with ASD, vmPFC *H* decreased with age (*r* = 20.31), but this was not observed in typical development (*r* = 0.18) (*F*_1,76_ = 5.1, *p* = .027, η^2^ = 0.06) (Figure 1A). Similarly, available longitudinal data (Figure 1B) showed a significant group × TP interaction, with vmPFC *H* decreasing in the ASD group but not in the TD group (*F*_1,23_ = 7.6, *p* = .01, η^2^ = 0.15).

No main effect of sex or group × sex interaction was observed in the cross-sectional analysis (Figure S1). The group × age interaction remained significant (*F*_1,68_ = 5.8, *p* = .018, η^2^ = 0.071) (Figure S2) in a male-only sample as well as when ASD children with a gestational age at birth, 36 weeks were excluded (*F*_1,68_ = 5.8, *p* = .018, η^2^ = 0.071) (Figure S3).

The group × age interaction also remained significant when the alternative (lower quality) fMRI acquisition and longitudinal data were included in a linear mixed-effects model (*t*_106_ = −2.37, *p* = .02).

### Reduced vmPFC *H* Is Associated With Within-DMN FC

Lower vmPFC *H* was associated with reduced FC within the DMN across groups (*F*_1,78_ = 4.8, *p* = .03, η^2^ = 0.06) (Figure 2). While the group × *H* interaction was not significant (*p* = .18), this effect seems to be primarily driven by the ASD group, with post hoc partial correlations (controlling for RMSD) conducted separately in each group showing a significant association between vmPFC *H* and within-DMN FC for the ASD group only (ASD: *r* = 0.40, *p* = .007; TD: *r* = 0.03, *p* = .800). Additionally, a significant group × age interaction was observed, with FC within the DMN decreasing with age in the ASD but not the TD group (*F*_1,78_ = 4.4, *p* = .038, η^2^ = 0.05), mirroring the group × age interaction observed for p32 *H*.

### Reduced vmPFC *H* Is Associated With Lower Emotional Control in Children With ASD

No significant relationship between ASD symptom severity (Autism Diagnostic Observation Schedule comparison score) and vmPFC *H* was observed (*r* = 0.007, *p* = .97). However, higher scores on the emotional control subscale of the BRIEF-Preschool version (indicative of greater problems with emotion regulation) were associated with a lower *H* in the vmPFC across groups (*r* = − 0.37, *p* = .03, partial correlation controlling for RMSD) (Figure 3A), with the same trend being observed in the ASD group only (*n* = 20; *r* = −0.38, *p* = .1) (Figure 3B).

## DISCUSSION

The current study found in cross-sectional as well as longitudinal analyses that the Hurst exponent estimated from natural sleep fMRI declined with age in the vmPFC (area p32) in a cohort of toddlers and preschoolers with ASD, while this was not observed in a matched group of TD children. These findings are consistent with evidence of lower vmPFC *H* observed later in the life span in ASD, in young adult males with ASD (25), suggestive of increased E/I ratio in autism. In our cohort, lower vmPFC *H* in toddlers and preschoolers was further associated with reduced FC within the DMN as well as with greater difficulties with emotional regulation (although this latter analysis was underpowered due to the BRIEF only being available in a subset of older preschoolers, given this measure’s norms and targeted age, and the finding was only significant in a combined ASD and TD sample). These results suggest an early onset of E/I imbalances in vmPFC in ASD with likely consequences for the maturation of the DMN and broader neurodevelopmental outcomes.

Unlike in Trakoshis *et al*. (25), we did not observe a diagnostic group × sex interaction for the vmPFC *H*. The most likely explanation for the absence of sex effects in our study is the relatively limited number of girls in the ASD cohort (which is, overall, consistent with the well-documented sex ratio in ASD prevalence). The group × age interaction remained significant when analyses were conducted in a male-only sample, suggesting that the inclusion of girls did not have a large effect on the results. Given the large gap in age between our cohort and the only other ASD cohort in which lower vmPFC *H* was reported (25), it is possible that sex differences in E/I balance become more prominent in puberty. For example, McSweeney *et al*. (36) observed a steeper decrease in the aperiodic exponent in healthy adolescent males than females in a longitudinal EEG study. In a study of TD 10- to 30-year-old individuals utilizing magnetic resonance spectroscopy, Perica *et al*. (37) also found the glutamate/GABA ratio to increase with age in the frontal cortex, particularly during adolescence. The authors concluded that this supports a critical period during adolescence for frontal cortex development. In the absence of studies spanning early childhood to adulthood in diverse populations, it is not possible to establish how differences in E/I balance in young children with ASD interact with changes in E/I balance that have been observed in adolescence and young adulthood. Furthermore, the underlying mechanisms that drive differences in E/I balance development remain largely unknown [see (16) for a review].

Our results suggest that the development of E/I balance in the vmPFC may already be disrupted as early as toddlerhood and preschool age in children with neurodevelopmental disorders such as autism. An early onset of E/I imbalances in children with autism is also supported by a recent study in 10-month-old infants with neurofibromatosis or a family history of neurodevelopmental disorders in whom a higher aperiodic exponent (derived from EEG) in combination with poorer attentional control at age 24 months predicted symptoms of autism at age 36 months (38). In another preliminary study in a small sample of 19 toddlers with ASD, lower aperiodic exponent determined from EEG collected in infancy was related to repetitive behaviors at 24 months (39). While these findings suggest early developmental alterations of the E/I ratio in autism, additional longitudinal research is needed to understand the spatial and temporal specificity and long-term consequences.

Our finding of an association between reduced within-DMN FC and reduced *H* in the vmPFC suggests that this region may be of particular importance—as well as being particularly vulnerable—in early brain development in children with ASD. Multiple studies have shown that vmPFC typically shows strong structural and FC with other regions of the DMN as well as the ventral striatum, amygdala, hippocampus, and thalamus (40–42). Reduced within-DMN FC compared with typical development has been reported consistently in studies of adolescents and adults with ASD [e.g., (26,43–45)]. In children with ASD, however, hyperconnectivity of the DMN has sometimes been observed. Padmanabhan *et al*. (26) hypothesized that a trajectory of early hyperconnectivity of the DMN in childhood to hypoconnectivity in adulthood in ASD may be due to a lack of neurotypical age-related increase in DMN FC. We did not find a main effect of diagnostic group for DMN FC in our cohort of toddlers and preschoolers. The observed positive correlation between vmPFC *H* and DMN FC (across groups) and the finding of decreasing vmPFC *H* with age in the ASD group (with the same pattern obvious in our smaller longitudinal sample) suggests that atypical development of E/I balance may precede reduced FC of the DMN in ASD.

In the current study, we extended results by Trakoshis *et al*. (25) by specifically focusing on an fMRI-derived estimate of E/I ratio in the vmPFC in a cohort of young children with ASD (note that area p32 was the only region to show significant effects after correction for multiple comparisons in their study). However, an altered developmental trajectory of E/I balance in ASD is unlikely to be specific to the vmPFC alone. In a supplementary analysis including all parcels from the HCP atlas (see Tables S4 and S5; Figure S4), multiple other regions showed significant (*p*, <.05, uncorrected) main effects of group and group × age interactions. The current study is underpowered to interpret these findings (with none of these results surviving adjustment for multiple comparisons at a Benjamini-Hochberg false discovery rate–adjusted *p*, <.05). Future research in independent cohorts is needed to better understand how this brainwide pattern of altered Hurst exponent development may be related to other patterns of structural and functional brain development [e.g., the sensorimotor-association axis (46) or expression of genes involved in E/I balance (47)].

Importantly, the current study was conducted during natural nocturnal sleep. E/I balance (48,49) and specifically the Hurst exponent (estimated from EEG) change between wakefulness and deep sleep stages in typical adults, particularly in the DMN (50). Therefore, it is possible that the reported results in part reflect differences in sleep in young children with ASD. Sleep problems are common in autism and neurodevelopmental disorders generally (51,52), but the impact of sleep problems on functional brain development in early childhood surprisingly remains not well understood. A recent study by our group in a partially overlapping cohort (33) directly tested the relationship between sleep problems and increased auditory thalamocortical FC [also observed during wakefulness in multiple autism studies across the life span, e.g., (53)] and observed that increased FC in toddlers and preschoolers with ASD scanned while asleep was associated with increased sleep latency. In the absence of simultaneous EEG, which is nearly impossible to acquire due to practical challenges of collecting both EEG and fMRI during natural sleep, it is not possible to conclusively determine whether children with ASD and their TD peers were in the same sleep stage during the fMRI acquisition or whether their sleep quality was comparable. However, to successfully collect high-quality fMRI data in this age group, a child (with or without ASD) must maintain deep, motionless sleep for the duration of the scan [also see supplementary analyses in (33)]. Therefore, we believe it is unlikely that the results reported here can be explained solely by systematic differences in sleep stage in the ASD and TD groups. Furthermore, the observed reduction of vmPFC *H* in young children with ASD is consistent with the findings in awake young adults with ASD by Trakoshis *et al*. (25), suggesting that it may be independent of wakefulness.

Lastly, the same methods and parameter settings used to estimate *H* in (25) were used for the current study despite the substantially different age range. The Hurst exponent can be estimated in several different ways [see e.g., (54) testing different approaches for fMRI data and impacts of factors such as scanner manufacturer on derived estimates], which makes comparisons across studies challenging. Therefore, this approach was chosen to keep the analyses as similar to those in Trakoshis *et al*. (25) as possible. While it is common to employ the same parameter settings when comparing neuroimaging results across ages [e.g., (55) estimated *H* from resting-state fMRI in adults ages 19–85 years], the temporal properties of the blood oxygen level–dependent signal vary substantially across the life span [e.g., (56)]. Simulations, multimodal longitudinal data (including magnetic resonance spectroscopy, for example), or studies in deeply sampled individuals are needed to establish how the Hurst exponent estimate reflects changes in E/I ratio during different physiological states and with age and how parameter settings may need to be adjusted to best capture these differences.

## Supplementary Material

Supplementary Material

Supplementary material cited in this article is available online at https://doi.org/10.1016/j.bpsc.2024.09.003.

## Figures and Tables

**Figure 1.**
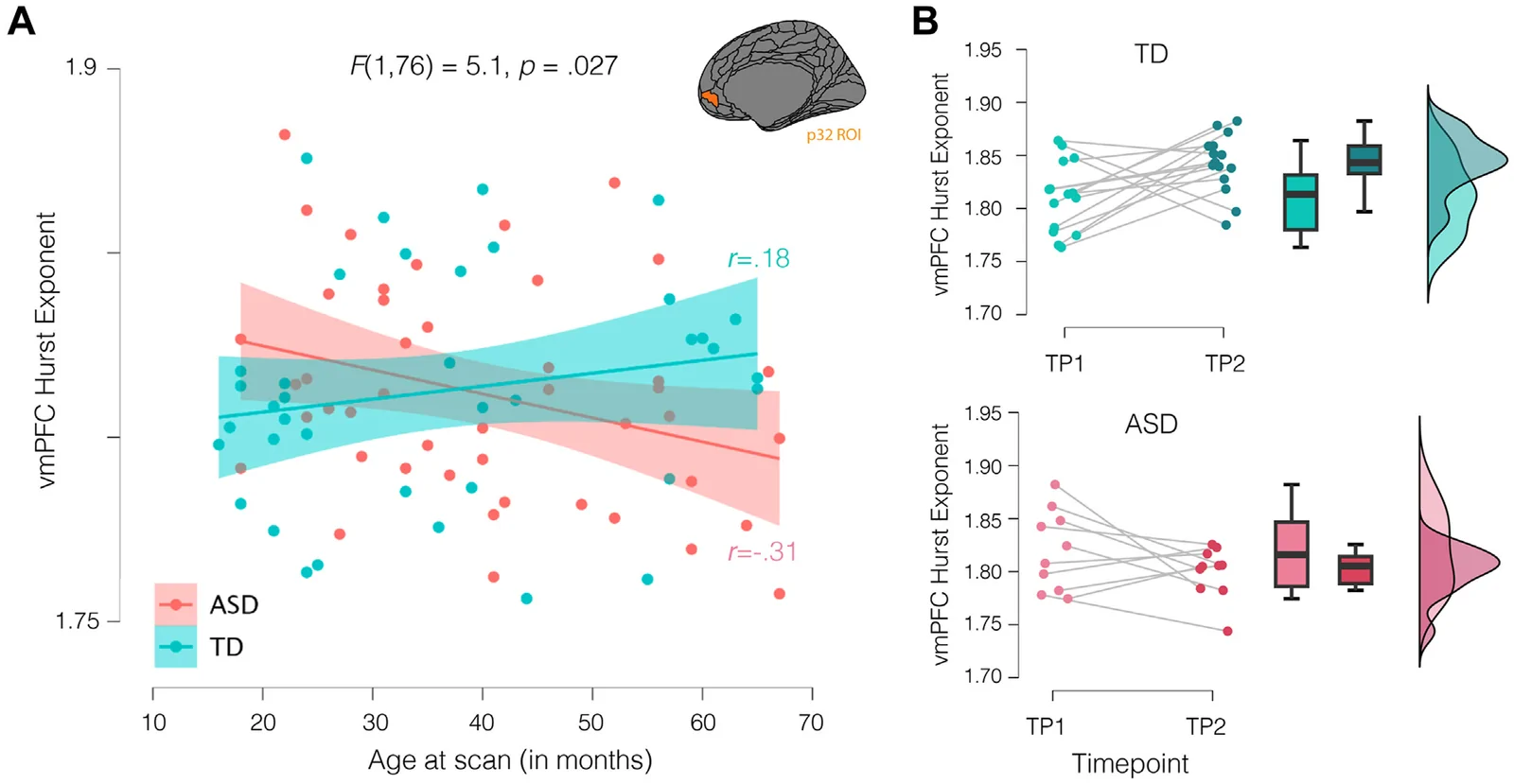
**(A)** The Hurst exponent *H* in the ventromedial prefrontal cortex (vmPFC) (area p32 as defined by the Human Connectome Project atlas, shown in the right corner) decreased with age in toddlers and preschoolers with autism spectrum disorder (ASD) (red) but not in age- and in-scanner motion–matched typically developing (TD) children (blue). Shaded areas show 95% CIs. The main effect of group was also significant (*F*_1,76_ = 4.7, *p* = .034, η^2^ = 0.05) but is not interpreted given the significant interaction. The main effect of sex (*F*_1,76_ = 0.02, *p* = .9, η^2^ = 0.00) and group 3 sex interaction (*F*_1,76_ = 0.03, *p* = .9, η^2^ = 0.00) were not significant and neither was the effect of root mean-square deviation (*F*_1,76_ = 3.25, *p* = .08). **(B)** The same pattern was observed within subjects in the subset of children with longitudinal functional magnetic resonance imaging acquisitions (average time between scans 26.3 months), with vmPFC *H* increasing in the TD group (top) but decreasing in children with ASD (bottom). The main effect of time point (TP) was not significant (*F*_1,23_ = 0.38, *p* = .54, η^2^ = 0.007) and neither was the effect of diagnostic group (*F*_1,23_ = 4.2, *p* = .052, η^2^ = 0.06). ROI, region of interest.

**Figure 2.**
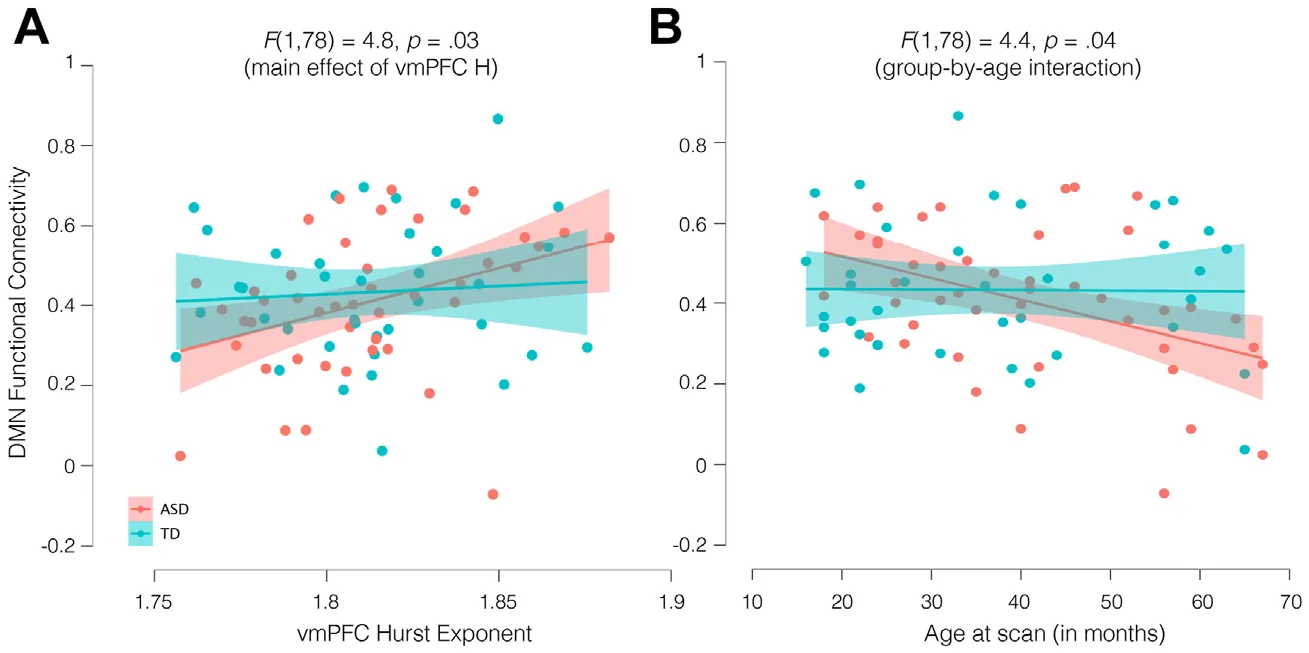
**(A)** Functional connectivity within the default mode network (DMN) correlates positively with the Hurst exponent *H* in area p32 (which is part of the DMN). **(B)** Functional connectivity in the DMN decreased with age in the autism spectrum disorder (ASD) but not the typically developing (TD) group (group × age interaction: *F*_1,78_ = 4.4, *p* = .04), mirroring the group × age interaction observed for p32 *H*. Note that the main effect of diagnostic group on DMN functional connectivity was not significant (*F*_1,78_ = 0.5, *p* = .48). vmPFC, ventromedial prefrontal cortex.

**Figure 3.**
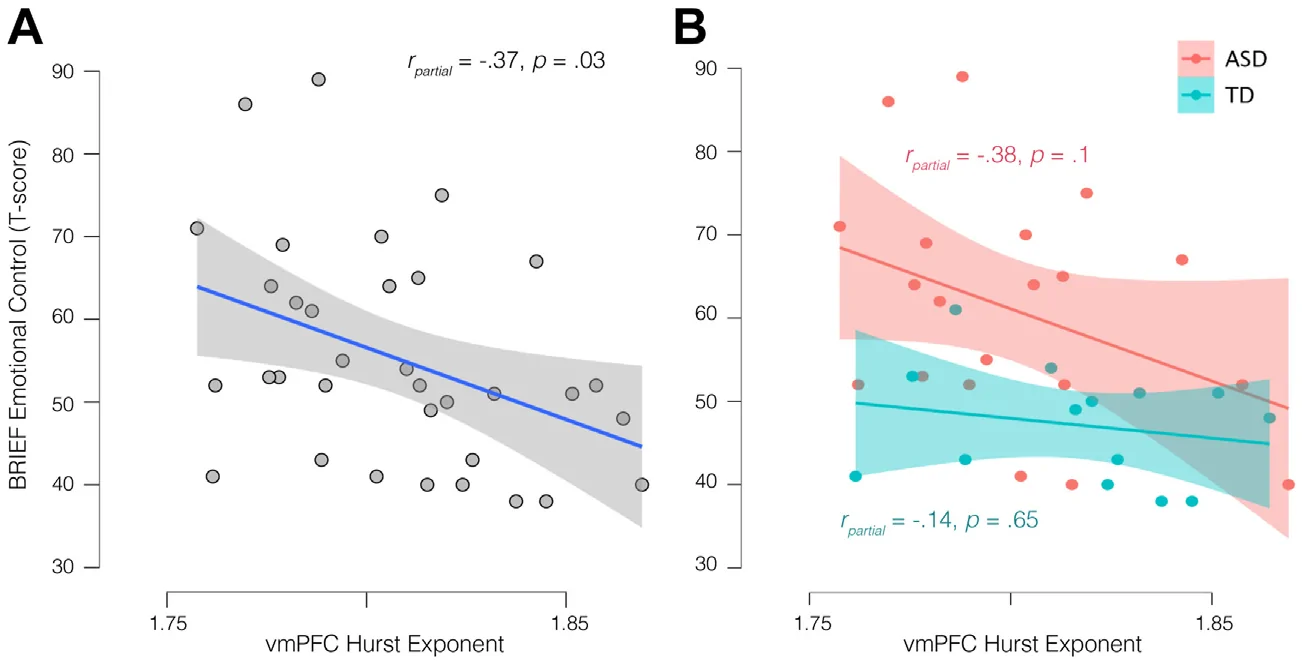
**(A)** Lower p32 *H* was associated with greater emotional dysregulation as assessed by the Behavior Rating Inventory of Executive Function (BRIEF)-Preschool version emotional control sub-scale. This correlation was assessed across diagnostic groups due to the BRIEF-Preschool version only having been administered to the subset of older children in the cohort (20 with autism spectrum disorder [ASD], 14 typically developing [TD]; mean age =50.1 months, SD = 9.56 months), in accordance with the targeted age range specified for this measure. **(B)** A post hoc analysis in both groups separately showed that this association was stronger (but not significant, *p* = .1) in the ASD group. vmPFC, ventromedial prefrontal cortex.

**Table 1.** Participant Characteristics for Children With High-Quality MRI Data

	ASD		TD		Group Comparisons, p Values		
	Female, *n* = 13	Male, *n* = 32	Female, *n* = 17	Male, *n* = 21	Diagnostic Group	Sex	Diagnostic Group × Sex
GA at Birth, Weeks	39.2 ± 1.2 [37–41]	38.3 ± 2.5 [31–43]	40 ± 1 [38–42]	39.4 ± 1.2 [37–42]	.04	.1	.7
Age at MRI Scan, Months	35.5 ± 15.5 [18–67]	42.2 ± 13.4 [24–67]	40.4 ± 14 [17–63]	33.6 ± 17.4 [16–65]	.6	.99	.06
Ethnicity, Hispanic or Latino, Yes/No	7/3	12/17	4/13	4/17	.01	.4	NA
Income-to-Needs Ratio	3.5 ± 3.5 [0.39–9.72]	3.2 ± 2.2 [0.25–6.75]	4.8 ± 3.1 [0–9.72]	4.0 ± 1.9 [0.33–6.75]	.12	.39	.77
Maternal Education Level	3.9 ± 2.0 [1–7]	3.8 ± 1.9 [1–7]	5.4 ± 1.4 [2–7]	5.2 ± 1.2 [2–6]	<.001	.76	.9
ADOS-2 Comparison Score	6.5 ± 2.8 [1–10]	6.3 ± 2.1 [2–10]	NA	NA	NA	.34	NA
RMSD, mm	0.15 ± 0.04 [0.08–0.18]	0.11 ± 0.03 [0.05–0.14]	0.10 ± 0.03 [0.05–0.17]	0.10 ± 0.03 [0.05–0.15]	.12	.6	.4

Values are presented as mean 6 SD [range] or n. GA at birth is based on caregivers’ report. INR is derived by dividing reported household income by the federal poverty level, adjusted for household size, with an INR of 1 corresponding to living at the federal poverty level. Maternal education level was coded on a 1 (incomplete high school) to 7 (advanced professional or doctoral degree) scale. Analyses of variance tested for main effects of diagnostic group and sex and diagnosis × sex interactions for GA at birth, age at MRI scan, in-scanner head motion (RMSD), INR, and maternal education level. A *t* test was used to test for sex differences in ASD symptom severity (ADOS-2 scores) in children with ASD only. Information on exact GA at birth was missing for 8 children with ASD (1 female, with 4 of 8 reported to be born at term) and 2 TD children (both reported to be born at term). Information on ethnicity (6 children with ASD), INR (5 TD children and 8 children with ASD), and maternal education level (4 children with ASD) was missing.

ADOS-2, Autism Diagnostic Observation Schedule-Second Edition; ASD, autism spectrum disorder; GA, gestational age; INR, income-to-needs ratio; MRI, magnetic resonance imaging; NA, not applicable; RMSD, root mean-square deviation; TD, typically developing.
