# Supplementary material for: Altered Development of the Hurst Exponent in the Medial Prefrontal Cortex in Preschoolers With Autism
Source: Biol Psychiatry Cogn Neurosci Neuroimaging. Author manuscript; Available in PMC 2026 Nov 4. (PMC13734569; doi:10.1016/j.bpsc.2024.09.003)
Supplement: Supplementary file 1 [file NIHMS2218116-supplement-Supplementary_Material.pdf]

SUPPLEMENTARY INFORMATION

Altered Development of the Hurst Exponent in Medial Prefrontal Cortex in Preschoolers With Autism

Linke *et al.*

Supplementary Materials

Exclusionary Criteria

Participants were excluded for ASD-related medical or genetic conditions (e.g., epilepsy, fragile X or Rett syndrome, tuberous sclerosis), or other neurological conditions (e.g., cerebral palsy, Tourette syndrome). Participants in the TD group were also excluded for premature birth (<36 weeks gestational age at birth), and personal or family history (first degree relatives) of ASD, intellectual disability, or other heritable psychiatric or neurological disorders.

Natural Sleep MRI

In preparation for the scan night, and to optimize MRI data acquisition, a comprehensive habituation protocol was implemented. After an individualized scan night sleep strategy (e.g., time of arrival, approximating home-like sleeping arrangements, including access to a double MRI bed for co-sleeping families, lighting in the MRI suite, etc.) was developed for each child, based on the typical bedtime routines and habits assessed in advance, families were instructed to practice inserting soft foam child-size earplugs and to play recordings of the MRI sequences employed in the study for at least a week prior to the scan. During the MRI session, children wore earplugs (~30 dB sound attenuation), and MRI-compatible headphones (MR Confon) were used to play white noise at a comfortable listening level to keep the noise level constant and prevent children from waking up during scan transitions. A weighted blanket kept children comfortable and reduced motion, and a caregiver and/or member of the research team remained in the scanner room throughout the session to monitor sleep, motion, and comfort.

MRI data preprocessing and denoising

The structural images were converted from dicom to nifti format and were coregistered to the mean functional images, segmented and normalized to MNI space using non-linear registration and the default tissue probability maps included with SPM12. The white matter (WM) probability maps obtained from segmentation of the structural image for each individual subject were thresholded at 0.95 and eroded by 1 voxel. WM and CSF time courses were extracted from the thresholded and eroded masks using aCompCor (Behzadi et al. 2003) for subsequent nuisance regression (see below).

Functional images were corrected for susceptibility-induced distortions using two 20s spin-echo EPI acquisitions with opposite phase encoding directions (and the same matrix size, FOV and prescription as the EPI scans) and FSL's TOPUP tools (Smith et al. 2005). Subsequently, functional images were motion-corrected using rigid-body realignment as implemented in SPM12. The Artifact Detection Toolbox (ART, as installed with CONN v17f) was used to identify outliers in the functional image time series from the resulting 6 motion parameters (3 translational and 3 rotational) that had frame-wise displacement (FD) >0.5 mm and/or changes in signal intensity that were greater than three standard deviations. As oscillations due to respiration are prominent in motion parameters derived from multiband EPI realignment (Fair et al., 2016) and would result in unnecessary censoring of large chunks of data in some participants, the thresholds to detect outliers were more lenient than those used for standard resting state fMRI acquisitions with slower TRs. To ensure that none of our findings were due to differences in apparent motion between groups, groups were matched on RMSD calculated from rigid-body realignment of the raw data prior to TOPUP correction (Table 1).

Functional images were directly normalized to MNI space with the same non-linear registration as used for the structural images. The MNI template rather than an age-appropriate template was used for normalization due to the wide age range and cross-sectional analyses conducted (see Chen et al. 2021 for a detailed discussion). Data were visually inspected for quality by two members of the research team (authors AL and BC) independently, at each pre-processing step, including determining successful normalization. Voxel timeseries were converted to percent-signal change separately for each EPI acquisition. Since all analyses were run on averaged voxel time series within pre-defined ROIs, no prior smoothing was applied to the data. Band-pass filtering using a temporal filter of 0.008 to 0.08 Hz was carried out as part of the nuisance regression which also included scrubbing of the motion outliers detected by the ART toolbox, and regression of the 6 motion parameters and their derivatives, as well as the first five PCA component time series derived from the CSF and white matter masks. The residuals of the nuisance regression were then used for all subsequent analyses.

To maximize sample size, only the best of the two fMRI runs was used in analyzes, allowing for inclusion of participants who woke up prior to completion of the second run or low data quality in one of the two runs (n=10). MRI datasets from 11 ASD participants were excluded from analyses due to excessive motion in both runs. The final ASD and TD groups with fMRI data did not differ on age, sex, inclusion of the first vs. second fMRI run, or head-motion during the fMRI scans (see Table 1).

Supplementary References

Behzadi Y, Restom K, Liau J, Liu TT. A component based noise correction method (CompCor) for BOLD and perfusion based fMRI. *Neuroimage*. 2007;37(1):90-101. doi:10.1016/j.neuroimage.2007.04.042

Chen, B., Linke, A., Olson, L., Ibarra, C., Kinnear, M., Fishman, I. Resting state functional networks in 1-to-3-year-old typically developing children. *Developmental Cognitive Neuroscience*. 2021; 51,100991. doi: 10.1016/j.dcn.2021.100991

Smith SM, Jenkinson M, Woolrich MW, et al. Advances in functional and structural MR image analysis and implementation as FSL. *NeuroImage*. 2004;23:S208-S219. doi:10.1016/j.neuroimage.2004.07.051

Fair DA, Miranda-Dominguez O, Snyder AZ, et al. Correction of respiratory artifacts in MRI head motion estimates. *NeuroImage*. 2020, Mar; 208: 116400. doi: 10.1016/j.neuroimage.2019.116400

Supplementary Tables

Table S1. Participant demographic and clinical characteristics for male-only cohort.

	ASD	TD	Group Comparison Statistics
n	32	21	
GA at birth (weeks)	38.3 ± 2.5 [31-43]	39.4 ± 1.2 [37-42]	*t*(43)=-1.7, *p*=.1
Age (months) at MRI scan	42.2 ± 13.4 [24-67]	33.6 ± 17.4 [16-65]	*t*(51)=2.0, *p*=.05
Ethnicity (yes/no Hispanic or Latino)	12/17	4/17	χ^2 = 2.8, *p*=.1
Income-to-needs ratio	3.2 ± 2.2 [0.25-6.75]	4.0 ± 1.9 [0.33-6.75]	*t*(44)=-1.4, *p*=.18
Maternal Education Level	3.8 ± 1.9 [1-7]	5.2 ± 1.2 [2-6]	*t*(49)=-2.9, *p*=.005
ADOS-2 SA	10.9 ± 4.6 [3-19]	n/a	n/a
ADOS-2 RRB	3.4 ± 2.2 [0-8]	n/a	n/a
ADOS-2 CSS (Comp. Score)	6.3 ± 2.1 [2-10]	n/a	n/a
RMSD	.11 ± .03 [.05-.14]	.10 ± .03 [.05-.15]	*t*(51)=.62, *p*=.5

Table S2. Participant demographic and clinical characteristics for cohort excluding children with ASD born < 36w gestational age.

	ASD		TD		Statistics		
	**Male**	**Female**	**Male**	**Female**	**Dx**	**Sex**	**Dx*Sex**
n	25	12	21	17	p = .27	n/a	n/a
GA at birth (weeks)	39.1 ± 1.7 [36-43]	39.2 ± 1.2 [37-41]	39.4 ± 1.2 [37-42]	40 ± 1 [38-42]	p = .13	p = .36	p = .47
Age (months) at MRI scan	42.0 ± 13.4 [24-67]	32.9 ± 12.9 [18-56]	33.6 ± 17.4 [16-65]	40.4 ± 14 [17-63]	p = .9	p = .74	p = .03
Ethnicity (yes/no Hispanic or Latino)	10/13	7/3	4/17	4/13	p = .007	p = .45	n/a
Income-to-needs ratio	2.9 ± 2.3 [0.3-6.8]	3.5 ± 3.5 [0.4-9.7]	4.0 ± 1.9 [0.3-6.75]	4.8 ± 3.0 [0-9.7]	p = .07	p = .29	p = .95
Maternal Education Level	3.6 ± 1.8 [1-6]	3.9 ± 2.0 [1-7]	5.2 ± 1.2 [2-6]	5.4 ± 1.4 [2-7]	p < .001	p = .56	p = .89
ADOS-2 SA	11.1 ± 4.7 [3-19]	12.0 ± 6.9 [0-19]	n/a	n/a	n/a	p = .63	n/a
ADOS-2 RRB	3.4 ± 2.1 [0-7]	2.8 ± 2.5 [0-8]	n/a	n/a	n/a	p = .39	n/a
ADOS-2 CSS (Comp. Score)	6.4 ± 2.1 [2-9]	6.5 ± 2.9 [1-10]	n/a	n/a	n/a	p = .91	n/a
RMSD	.10 ± .03 [.05-.14]	.11 ± .04 [.08-.18]	.10 ± .03 [.05-.15]	.10 ± .03 [.05-.17]	p = .3	p = .55	p = .35

Table S3. Participant characteristics for longitudinal cohort.

	ASD		TD			
	TP1	TP2	TP1	TP2	TP1	TP2
n	10		15			
# female	4		4		*p* = .48	n/a
GA at birth (weeks)	38.5 ± 1.35 [35-40]		39.3 ± 1.54 [39-43]		*p* = .18	n/a
Age (months) at MRI scan	28.3 ± 12.3 [18-52]	52.9 ± 11.5 [38-65]	25.0 ± 7.7 [18-45]	50.6 ± 12.5 [38-66]	*p* = .11	*p* = .65
Time between scans (months)	21.2 ± 8.36 [12-39]		29.7 ± 11.5 [16-46]		n/a	*p* = .06
RMSD	.11 ± .04 [.08-.17]	.1 ± .04 [.05-.16]	.11 ± .03 [.05-.17]	.12 ± .06 [.05-.24]	*p* = .15	*p* = .19

Table S4. Other HCP parcels that showed significant ($p<.05$, uncorrected) main effects of group (also see Figure S4B).

Region	Hemisphere	ASD mean	TD mean	t-value	p-value
s32	right	1.822	1.803	-2.887	0.005
LO2	right	1.818	1.802	-2.840	0.006
IFJa	right	1.821	1.820	-2.728	0.008
VMV3	left	1.805	1.817	2.693	0.009
5L	right	1.838	1.817	-2.568	0.012
V3B	left	1.813	1.819	2.495	0.015
OP1	right	1.819	1.811	-2.476	0.015
V3A	left	1.810	1.807	-2.261	0.027
IP0	left	1.824	1.821	2.231	0.029
A1	right	1.815	1.792	-2.219	0.029
5L	left	1.830	1.821	-2.190	0.031
PH	left	1.827	1.837	2.159	0.034
V4t	left	1.821	1.817	2.152	0.035
p24pr	left	1.814	1.815	-2.147	0.035
IFSp	right	1.818	1.828	-2.147	0.035
47s	left	1.809	1.805	-2.108	0.038
p24pr	right	1.828	1.817	-2.093	0.040
PIT	right	1.820	1.810	-2.053	0.043
31a	left	1.815	1.823	2.051	0.044
PoI2	left	1.822	1.829	2.048	0.044
V4t	right	1.818	1.813	-2.018	0.047

Table S5. Other HCP parcels that showed significant (p<.05, uncorrected) main group-by-age interactions (also see Figure S4C). Positive t-values reflect decreasing Hurst Exponent with age in the ASD but not TD group like observed for region p32.

Region	Hemisphere	t-value	p-value
IFJa	right	2.849	0.006
IFSp	right	2.704	0.008
d32	right	-2.460	0.016
p24pr	left	2.450	0.017
IP0	left	-2.435	0.017
V4t	left	-2.428	0.018
V3B	left	-2.406	0.018
VMV3	left	-2.386	0.019
47l	left	2.361	0.021
PGs	right	2.326	0.023
LO2	right	2.289	0.025
s32	right	2.237	0.028
PHT	left	-2.236	0.028
24dv	left	2.223	0.029
25	left	2.160	0.034
8BL	left	2.158	0.034
V3A	left	2.152	0.035
PFcm	left	2.147	0.035
OP1	right	2.128	0.036
PFcm	right	2.081	0.041
47s	left	2.061	0.043
LO1	right	2.054	0.043
5L	left	2.029	0.046

Supplementary Figures

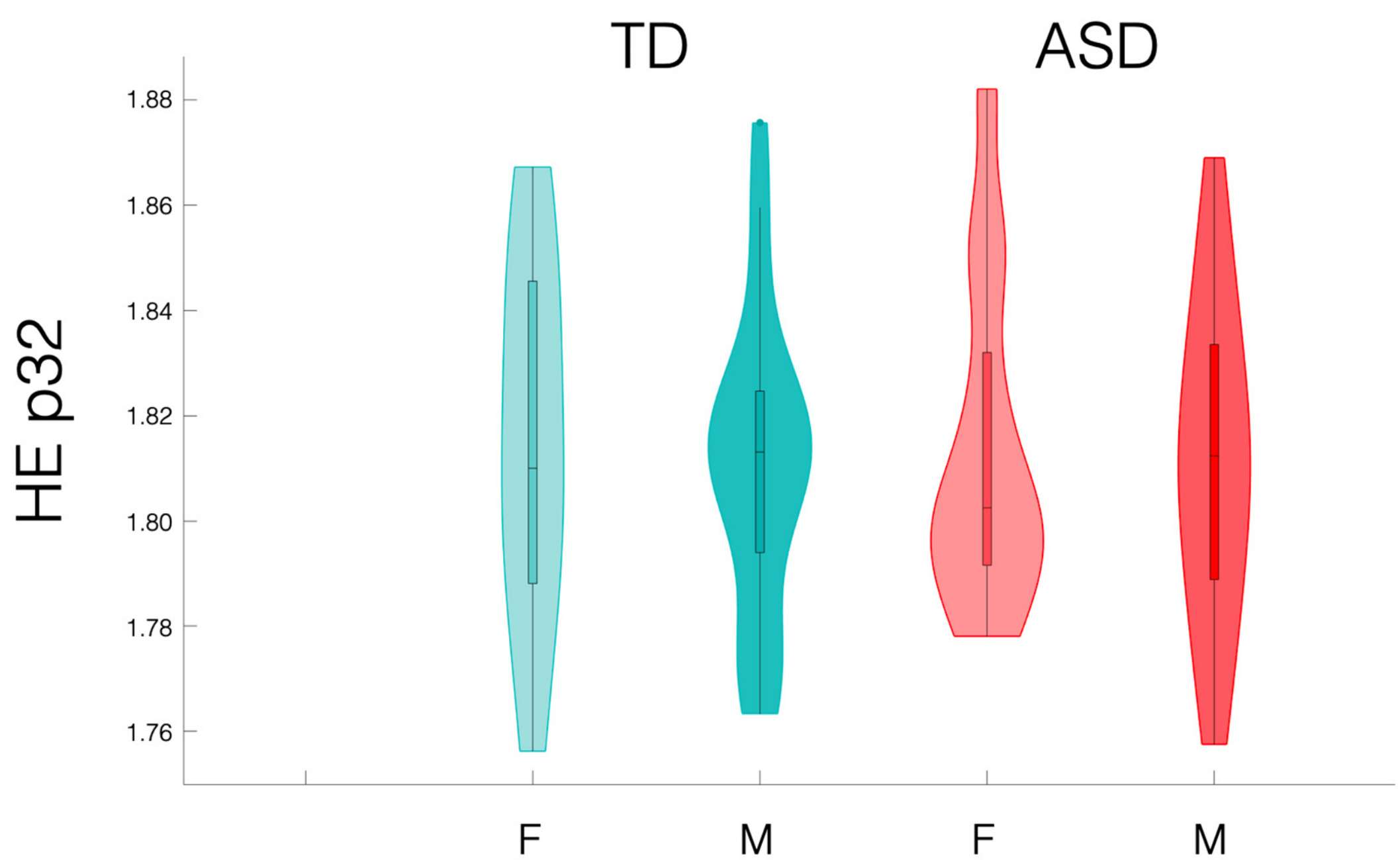

Figure S1. No significant interaction between sex and diagnosis for p32 Hurst Exponent.

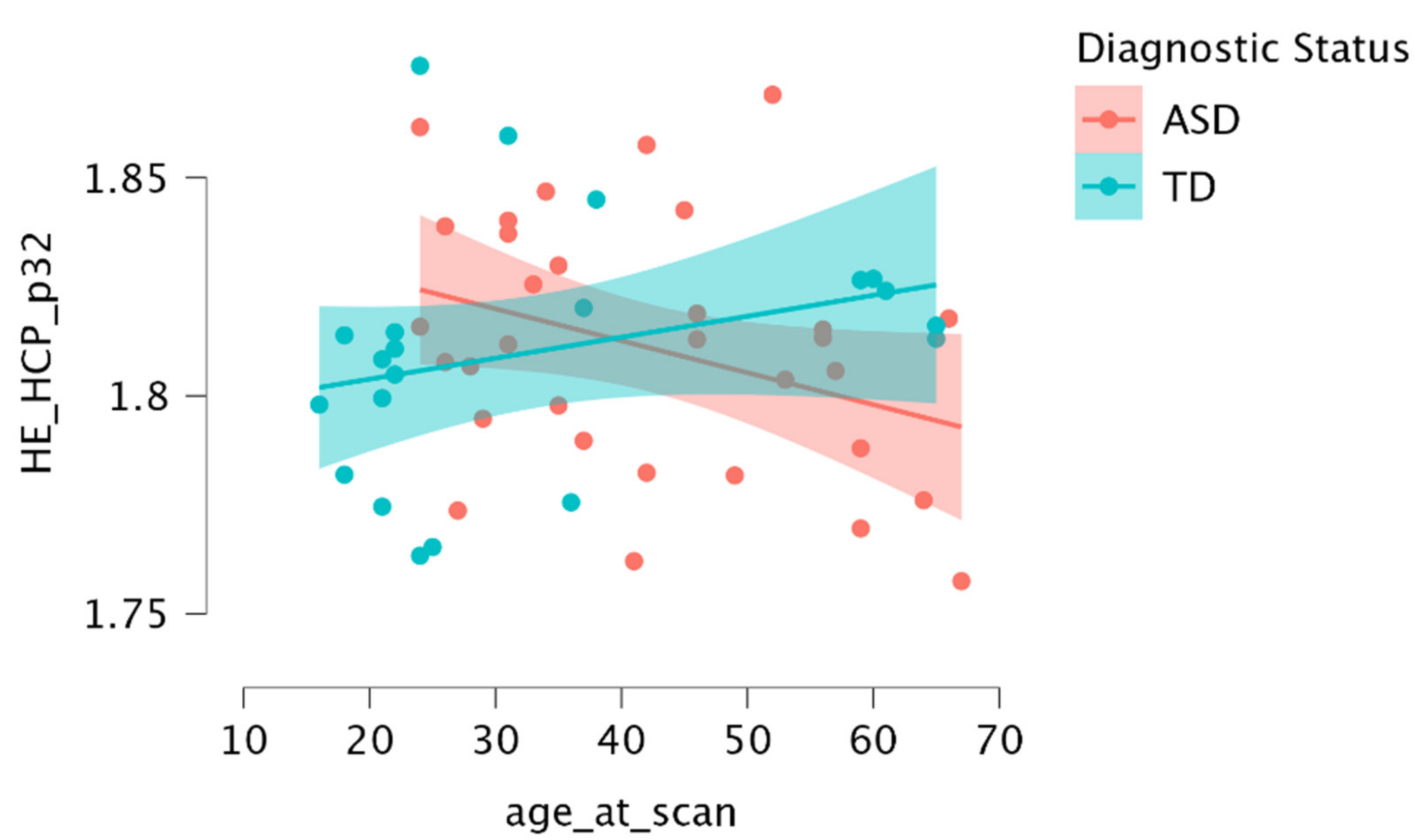

Figure S2. Diagnosis-by-age interaction effect on p32 HE for male-only cohort

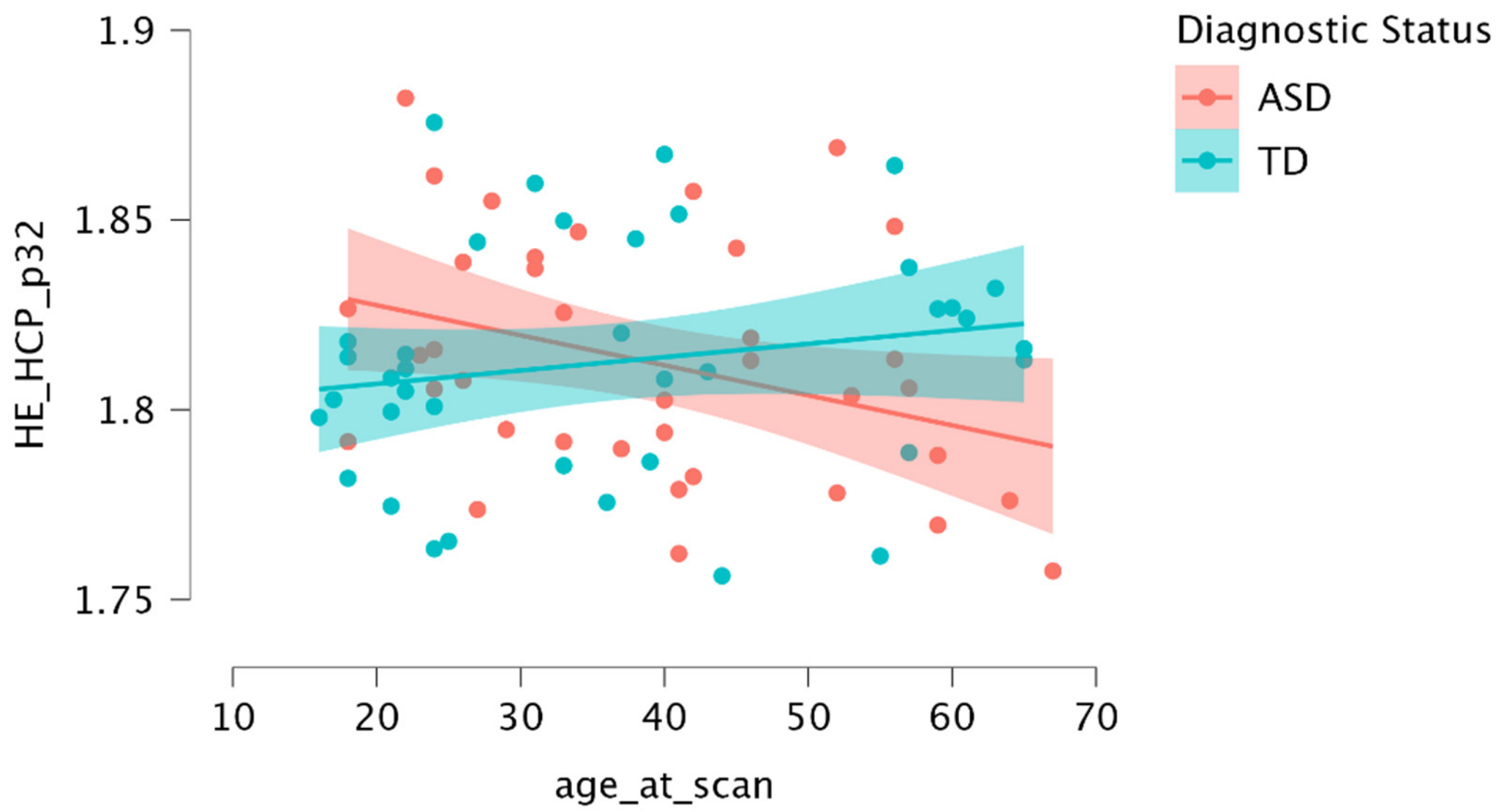

Figure S3. Diagnosis-by-age interaction effect on p32 HE when excluding children <36w gestational age.

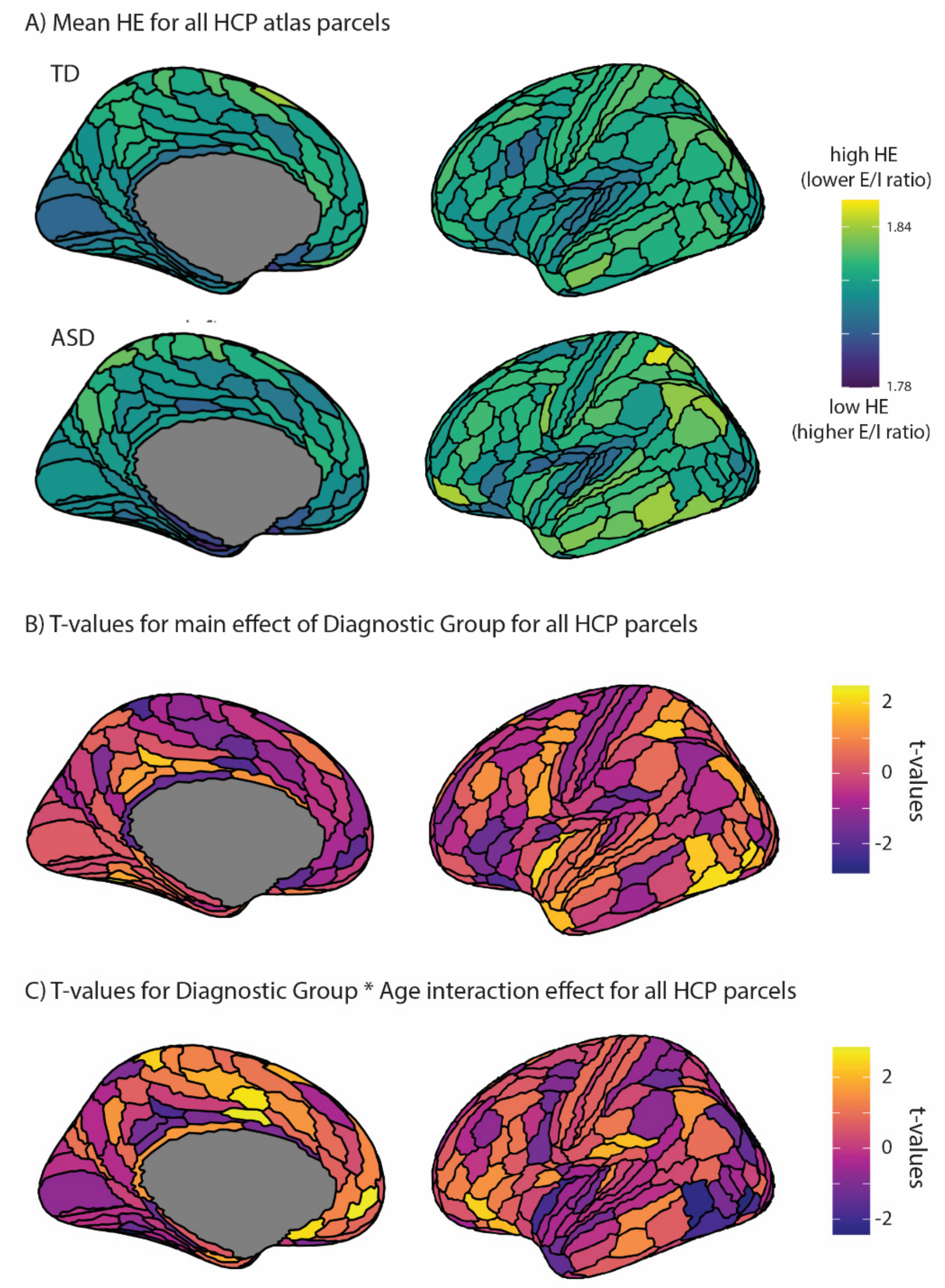

Figure S4. A) Whole-brain map of mean HE values for each HCP atlas parcel for the TD (top) and ASD (bottom) groups. B) T-values for the main effect of diagnostic group for each HCP atlas parcel (ASD>TD contrast, i.e. regions with positive t-values have higher mean HE in the ASD than TD group while regions with negative t-values have lower HE in the ASD compared to the TD group). C) T-values for the Diagnostic Group * Age interaction effect. Positive values indicate decreasing HE with age in the ASD group but increasing HE with age in the TD group.
